# Comparative Efficacy of iPACK vs Popliteal Sciatic Nerve Block for Pain Management Following Total Knee Arthroplasty: A Retrospective Analysis

**DOI:** 10.7759/cureus.51557

**Published:** 2024-01-03

**Authors:** Francisco Teixeira, Cristina P Sousa, Ana Patrícia Martins Pereira, Delilah Gonçalves, José C Sampaio, Miguel Sá

**Affiliations:** 1 Anesthesiology Department, Centro Hospitalar Trás-os-Montes e Alto Douro, Vila Real, PRT; 2 Anesthesiology Department, Centro Hospitalar Trás-os-montes E Alto Douro, Vila Real, PRT

**Keywords:** postoperative pain, continuous adductor canal block, ipack block, sciatic nerve block, regional anesthesiology, acute pain, primary total knee arthroplasty

## Abstract

Introduction

Total knee arthroplasty (TKA) is associated with severe acute postoperative pain. The use of tourniquets and drains (T/D) is common in TKA but may have an influence on postoperative pain and muscular strength. The infiltration of local anesthetic between the popliteal artery and capsule of the knee (iPACK block) is a motor-sparing block that provides analgesia to the posterior aspect of the knee. However, evidence regarding its efficacy is scarce. This study aims to assess the effectiveness of iPACK block and the impact of T/D use on pain and muscular strength after TKA.

Material and methods

A retrospective study was carried out including patients who underwent TKA from January 2020 to April 2023. Patients were allocated into groups according to the peripheral nerve block performed and T/D use.

Results

We included 415 patients in this study. No differences were found in pain at rest or the need for rescue analgesia between patients who received an iPACK block or sciatic nerve block (SNB) with T/D applied. Patients who received a SNB reported lower pain scores on movement (*p* = 0.019), but with a higher prevalence of motor block (p < 0.001). Patients who underwent surgery without using T/D reported lower pain scores on movement (*p* = 0.021) and reduced need for rescue analgesia (*p *= 0.041).

Conclusion

These findings indicate that iPACK block can facilitate early mobilization after TKA without significant impact on postoperative muscle strength. Furthermore, the use of a T/D may be a source of postoperative pain that could compromise early rehabilitation.

## Introduction

Total knee arthroplasty (TKA) is performed in patients with advanced-stage osteoarthritis when conservative treatment fails [1]. TKA is associated with severe acute postoperative pain, interfering with early ambulation and adherence to rehabilitation programs [1]. About 20% of patients may experience chronic postoperative pain, its main predictive factor being the presence of inadequately controlled acute postoperative pain [2]. Additionally, one in five will experience persistent (> 3 months) postoperative opioid use [3].

Pain management is a crucial aspect of patient care. Peripheral nerve blocks are recommended for the pain management of patients undergoing TKA, allowing better pain control and adherence to fast-track surgery programs [4]. The key to early recovery in fast-track TKA management lies in an ideal analgesic regimen that effectively manages postoperative pain while preserving motor function, enabling early mobilization.

The adductor canal block (ACB) is a regional anesthesia technique used to provide analgesia to the anterior and medial areas of the thigh and the knee [5]. The ACB targets the nerves that pass through the adductor canal. The primary nerve target of the ACB is the saphenous nerve, a branch of the femoral nerve. The saphenous nerve is responsible for the sensitive innervation of the leg’s medial section, from the knee to the ankle. In addition to the saphenous nerve, the ACB may also block the anterior branches of the obturator nerve and the medial femoral cutaneous nerve, which supply sensation to the medial section of the thigh [6]. It is important to note that the ACB does not provide complete analgesia to the knee joint, as the articular branches of the obturator nerve and the genicular branches of the tibial and common peroneal nerves are not targeted with this technique. Although the innervation to the vastus medialis travels within the region of the adductor canal (most likely in its own fascial tunnel), the ACB block shows a lower incidence of quadriceps muscle weakness compared to the femoral nerve block [7]. The ACB has been shown to provide effective analgesia for knee surgeries, particularly when combined with other regional anesthesia techniques for the posterior aspect of the knee [1].

Sciatic nerve block (SNB) at the popliteal level results in anesthesia of the lower limb below the knee, except for the medial leg and foot [5]. The motor fibers of the hamstring muscles are spared in this approach. However, while the SNB theoretically results in the best analgesia for the knee’s posterior area, motor weakness of the lower extremity compromises early rehabilitation programs. The iPACK block (infiltration of local anesthetic between the popliteal artery and capsule of the knee) is a motor-sparing block that targets the sensitive articular branches of the sciatic and obturator nerves, providing analgesia to the posterior section of the knee while sparing the motor branches of the tibial and common peroneal nerves [5,8]. Therefore, the iPACK block emerges as an alternative analgesic supplement to the ACB for posterior knee pain after TKA.

The use of tourniquets and postoperative surgical drains (T/D) is a common practice in TKA. In recent years, there has been a growing debate about the risks of tourniquet and drainage as their real benefits are not clear. Tourniquet application is expected to improve surgery exposure and cementation process in TKA, allowing a better cement penetration with a superior initial fixation strength and potentially reducing the long-term risk of aseptic loosening of the implant [9]. However, tourniquet use may also have some influence on motor strength, postoperative pain, and neurologic complications [10]. The purpose of not using a tourniquet during surgery is to improve blood flow to the area, which can lead to reduced pain or swelling, faster healing, and better surgical hemostasis [11]. Postoperative drainage in TKA is intended to reduce hematoma and swelling around the surgical site. There is insufficient conclusive evidence supporting the benefits of drainage use, as indicated by studies such as Basilico et al. In fact, their findings suggest that the employment of drains did not result in advantages concerning the reduction of pain, swelling, or hematoma [12].

This study primarily aims to compare the analgesic effectiveness of the iPACK block with the SNB at popliteal level as an alternative approach for pain management and motor preservation in TKA. Also, in this study, we evaluated if the usage of T/D had any impact on the analgesic efficacy and motor function as a secondary outcome.

## Materials and methods

**Figure 1:**
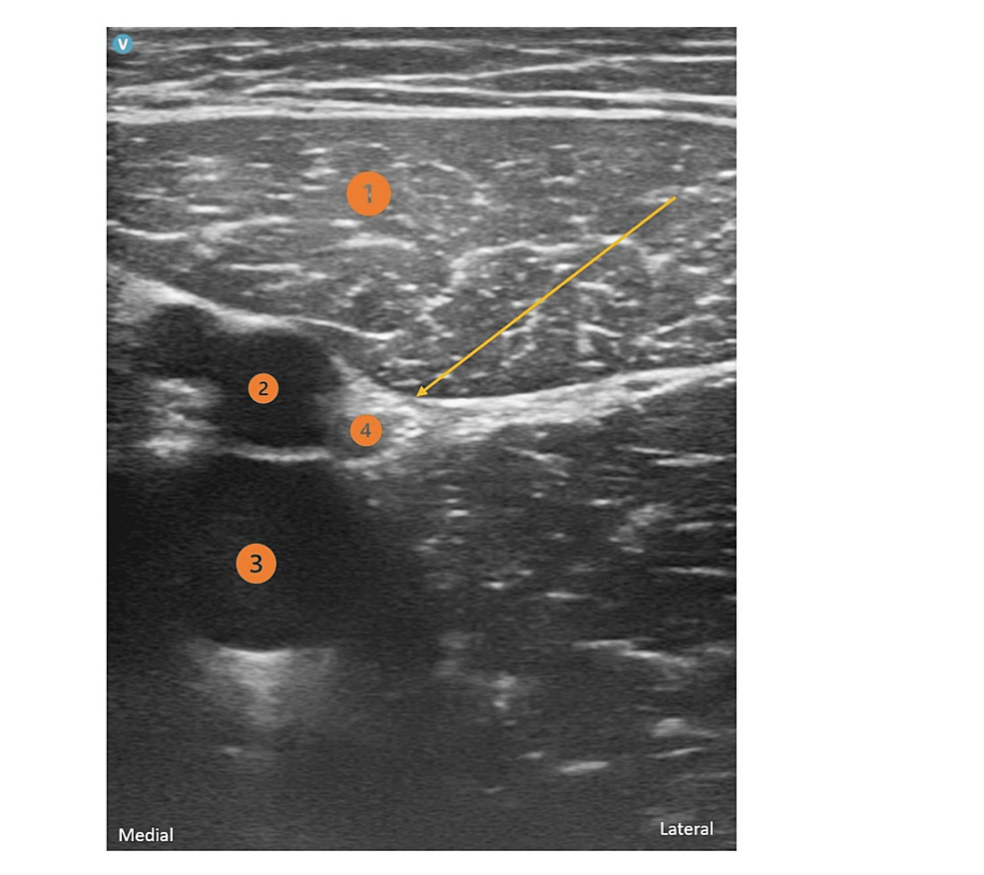
Adductor Canal Block 1) Sartorius muscle; 2) femoral artery; 3) femoral vein; 4) saphenous nerve Yellow arrow: Needle direction and target point

**Figure 2:**
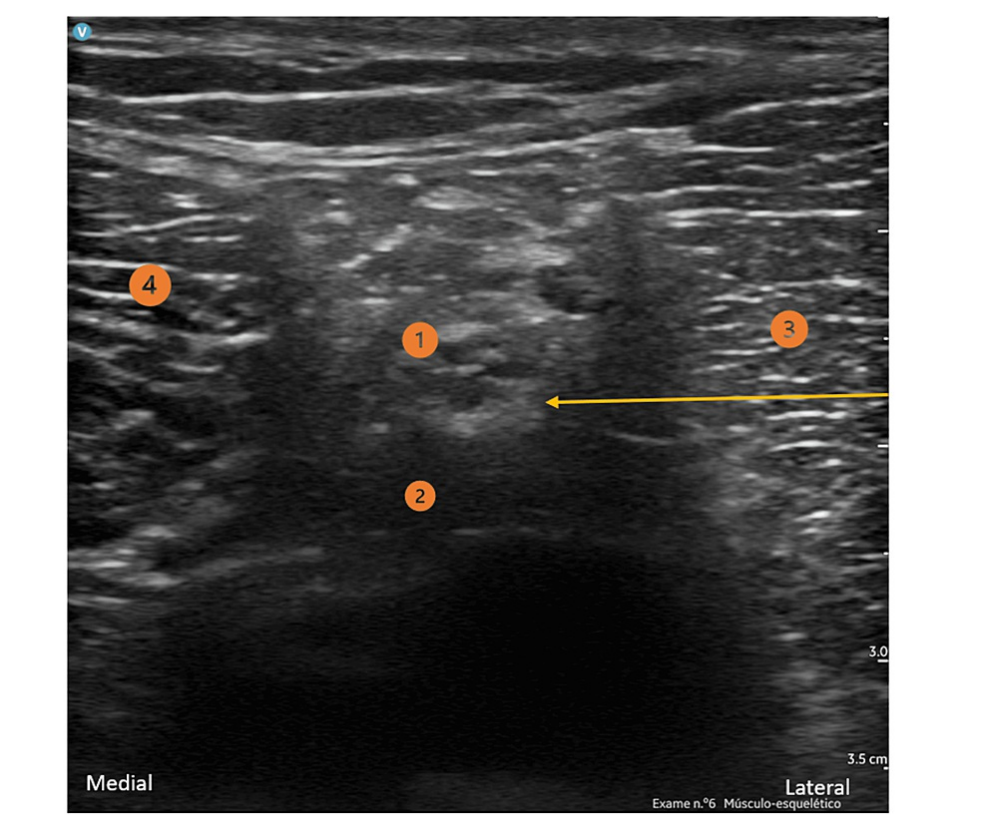
Sciatic Nerve Block (popliteal approach) 1) Sciatic nerve; 2) popliteal artery; 3) biceps femoris muscle; 4) semitendinosus Yellow arrow: Needle direction and target point

**Figure 3:**
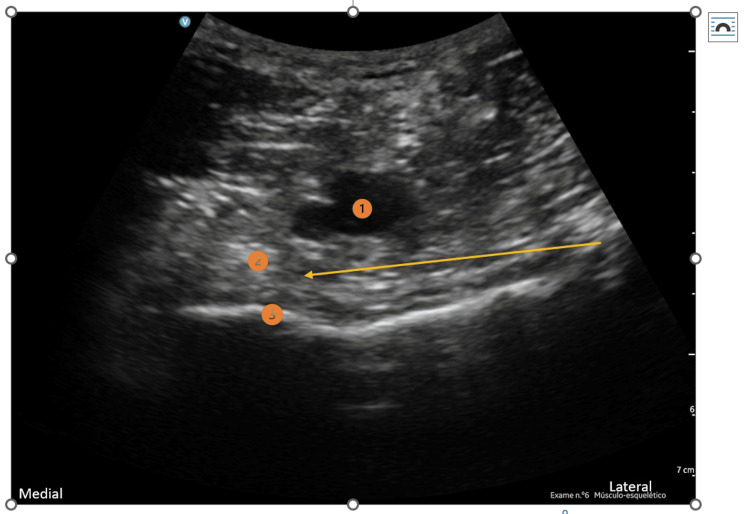
IPACK block 1) Popliteal artery; 2) interspace between the popliteal artery and posterior knee capsule; 3) femoral shaft Yellow arrow: Needle direction and target point

After institutional ethics committee approval, a retrospective study was carried out including patients who underwent TKA from January 2020 to April 2023 (Approval number: 133/2023 - CES). All patients received peripheral nerve blocks as part of a multimodal analgesic strategy and the procedure was conducted under spinal anesthesia. Patients under 18 years old, patients with previous surgery on the same knee, and patients undergoing general anesthesia were excluded from this work. Cases involving unsuccessful blocks, malfunctioning perineural catheters, or catheter exteriorization before the 48-hour postoperative period were also excluded from this study. In 2020, patients undergoing TKA received continuous ACB (cACB) (Figure 1) and a popliteal approach SNB (Figure 2) or an iPACK block (Figure 3), and the surgery was performed with the application of a tourniquet and surgical drains. Since 2021, our institution’s anesthesiology department, in collaboration with the orthopedic and rehabilitation services, tried to implement a strategy focused on improving post-surgery recovery for patients undergoing TKA. According to this strategy, patients scheduled for TKA received a cACB along with an iPACK block (at the level of the superior border of the femoral condyle) before surgical incision. During surgery, the tourniquet was only inflated prior to the implantation of the prosthetic material and the use of surgical drains was omitted, that is without T/D. However, the decision to follow the referred strategy was the orthopedic and anesthesiology team's responsibility.

Therefore, during the study period, patients could be divided into three groups depending on the strategy used. In Group A, patients received a cACB along with a single-shot SNB, and surgical procedures involved the use of T/D. In Group B, patients received a cACB and a single-shot iPACK block, but surgeries were conducted without using T/D. In Group C, patients received a cACB and a single-shot iPACK block, and surgeries were performed using T/D. It should therefore be noted that patients who received SNB always underwent surgery with a T/D. Only patients undergoing primary arthroplasty were included in this study.

In the perioperative period, patients were monitored concordantly with the ASA standards. Spinal anesthesia was performed using 10 mg 0.5% hyperbaric bupivacaine and sufentanil 0,002 mg at the L3-4 or L4-5 intervertebral space. After spinal anesthesia onset, cACB plus SNB or cACB plus iPACK block were performed.

To perform the peripheral nerve blocks, an ultrasound-guided (GE HealthCare® LOGIQ™ P9 XDclear™ Ultrasound Machine) in-plane needling technique was used. For the iPACK block, the transducer was placed over the popliteal fossa crease and the tibial nerve, common peroneal nerve, popliteal vessels, and femoral condyles were identified. From this location, the probe was slid proximally until the flat surface of the femoral shaft was visible. The needle was then inserted in-plane, from the lateral side of the knee, toward the space between the popliteal artery and the femur. The local anesthetic solution (10-15 ml of 0.5% ropivacaine) was injected into the popliteal artery’s anterior space. For the ACB, a linear probe was placed at the mid-thigh to identify the saphenous nerve, which is usually located anterolateral to the femoral artery and surrounded by the sartorius muscle anteriorly, the vastus medialis muscle anterolaterally and the adductor longus muscle posteromedially. The proximal limits of the adductor canal (or the apex of the femoral triangle) were identified through the proximal and distal scanning of the thigh to find the point where the medial border of the sartorius muscle meets the medial border of the adductor longus muscle. The needle was then inserted in-plane in a latero-medial direction, advanced toward the lateral border of the superficial femoral artery, and 10 ml of 0.2% ropivacaine was injected. Subsequently, a perineural catheter (Pajunk® SonoLong Echo 19G 50 mm Facet Tip needle and a 20G 50 cm catheter) was inserted 3 cm beyond the needle tip in the adductor canal for postoperative analgesia. For the popliteal SNB, a linear probe was placed in a transverse position at the popliteal crease, and the sciatic nerve sheath containing both components of the sciatic nerve (tibial and common peroneal nerves) was identified. An in-plane approach was used and 15 ml of 0.5% ropivacaine was injected.

In the peri-operative period, all patients received 4 or 8 mg of IV dexamethasone after skin incision and 10 mg/kg of IV tranexamic acid (with the maximum dose of 1 g) if there were no contraindications. The dose of dexamethasone administered, 4 mg or 8 mg, was at the attending anesthesiologist’s discretion. Also, systemic analgesia was performed with parecoxib 40 mg IV (adjusted for renal function) before tourniquet application (if used) and acetaminophen 1 g IV. A multimodal analgesia regimen was prescribed for the 48 h postoperative period, including acetaminophen 1 g IV 6 hourly, parecoxib 40 mg IV 12 hourly or ketorolac 30 mg IV 8 hourly, and a continuous perineural infusion of 250 ml of ropivacaine 0,2% at a rate of 5.2 ml/h. The postoperative analgesic plan included the rescue administration by the nursing staff, with consent of the attending physician, of 5 ml perineural ropivacaine 0.2% with 2-hour intervals, if the patients reported pain in the anterior region of the knee, or morphine 2 mg IV, if the pain was reported in the posterior area of the knee or if the pain was refractory to the perineural ropivacaine administration.

The analgesic efficacy was measured using a qualitative pain scoring system [13] (“no pain” = 0; “slight pain” = 1; “moderate pain” = 2; “intense pain” = 3; “unbearable pain” = 4) at rest (R) or with movement (M) (knee flexion and extension), and the need for rescue analgesia. Patients were assessed by the Acute Pain Unit team, which included an anesthesiologist and a nurse, at 24 and 48 hours after surgery. The maximum pain and minimum pain scores at rest and on movement reported during the 48 hours of follow-up were recorded. The Bromage score [14] (“absence of motor block” =0; “slight decrease in muscle strength” =+; marked decrease in muscle strength=++) was used to assess motor blockade, namely the ability of plantar and toe flexion (tibial nerve), as well as ankle dorsiflexion and toe extension (peroneal nerve). Again, the maximum motor blockade observed during the follow-up period was recorded.

Comparisons between groups were made using the Pearson's chi-square test. The significance level for all results was defined as p < 0.05. Data analysis was performed using the SPPS 29 program (IBM Corp. Released 2022. IBM SPSS Statistics Version 29.0. Armonk, NY: IBM Corp).

## Results

**Figure 4:**
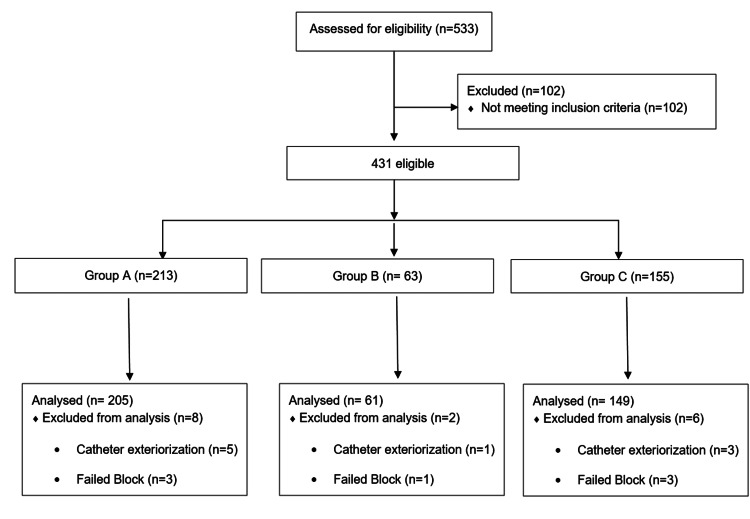
Consort flowchart

Among the 533 patients that were submitted to TKA from January 2020 to April 2023, 415 patients were properly enrolled and subjected to analysis (Figure 4). Two hundred and five patients were allocated to Group A (cACB along with a single-shot SNB, surgical procedures involved the use of T/D), 61 to Group B (cACB and a single-shot iPACK block, surgeries without using T/D), and 149 to Group C (cACB and a single-shot iPACK block, surgeries were performed using T/D), according to the performed anesthetic and surgical techniques.

Analyzing Table 1, gender distribution and age were similar between the groups. No significant differences were found in the pain scores at rest or in the need for rescue analgesia between the groups that received iPACK block (Group C) or SNB (Group A) with T/D applied. However, we found a statistically significant difference (p = 0.019) in pain at movement, showing better scores for patients in Group A. A higher prevalence of motor block was also observed in this group (p < 0.001).

**Table 1:** Demographic characteristics and comparison between Group A and Group C. M: motor; R: rest; N: number; *: statistically significant; +: slight decrease in muscle strength; ++: marked decrease in muscle strength.

		Group A	Group C	
				p-value
N		205	149	
Sex F/M N (%)		144 (70.2%)/61 (29.8%)	109 (73.2%)/40 (26.8%)	p = 0.549
Age (years) N (%)				p = 0.679
	40-49	2 (1.0%)	1 (0.7%)	
	50-59	30 (14.6%)	15 (10.1%)	
	60-69	73 (35.6%)	55 (36.9%)	
	70-79	82 (40.0%)	62 (41.6%)	
	80-89	18 (8.8%)	15 (10.1%)	
	³ 90	0 (0.0%)	1 (0.7%)	
Maximum pain R N (%)				p = 0.277
	0	123 (60.0%)	83 (55.7%)	
	1	74 (36.1%)	55 (36.9%)	
	2	8 (1.0%)	9 (6.0%)	
	3	0 (0.0%)	2 (1.3%)	
	4	0 (0.0%)	0 (0.0%)	
Maximum pain M N (%)				p = 0.019*
	0	24 (11.7%)	6 (4.0%)	
	1	107 (52.2%)	70 (47.0%)	
	2	65 (31.7%)	64 (43.0%)	
	3	9 (4.4%)	9 (6.0%)	
	4	0 (0.0%)	0 (0.0%)	
Rescue analgesia N (%)		30 (14.6%)	26 (17.4%)	p = 0.474
Motor blockade N (%)				p < 0.001*
	0	149 (72.7%)	124 (83.2%)	
	+	45 (22.0%)	24 (16.1%)	
	++	11 (5.4%)	1 (0.7%)	

The results of the comparative analysis between Group B and Group C are exposed in Table 2. A significant difference was found in pain scores at movement (p = 0.021) and in the need for rescue analgesia (p = 0.041), showing lower pain scores and lower need for rescue analgesia in Group B. No differences were found regarding the Bromage scale scores.

**Table 2:** Demographic characteristics and comparison between Group B and Group C. M: motor; R: rest; N: number; *: statistically significant; +: slight decrease in muscle strength; ++: marked decrease in muscle strength.

		Group B	Group C	
				p-value
N		61	149	
Sex (F/M)		45 (73.8%)/16 (26.2%)	109(73.2%)/40 (26.8%)	p = 0.927
Age (years)				p = 0.567
	40-49	1 (1.6%)	1 (0.7%)	
	50-59	5 (8.2%)	15 (10.1%)	
	60-69	24 (39.3%)	55 (36.9%)	
	70-79	29 (47.5%)	62 (41.6%)	
	80-89	2 (3.3%)	15 (10.1%)	
	>90	0 (0.0%)	1 (0.7%)	
Maximum pain R N (%)				p = 0.158
	0	43 (70.5%)	83 (55.7%)	
	1	17 (27.9%)	55 (36.9%)	
	2	1 (1.6%)	9 (6.0%)	
	3	0 (0.0%)	2 (1.3%)	
	4	0 (0.0%)	0 (0.0%)	
Maximum pain M N (%)				p = 0.021*
	0	8 (13.1%)	6 (4.0%)	
	1	31 (50.8%)	70 (47.0%)	
	2	22 (36.1%)	64 (43.0%)	
	3	0 (0.0%)	9 (6.0%)	
	4	0 (0.0%)	0 (0.0%)	
Rescue analgesia N (%)		4 (6.6%)	26 (17.4%)	p = 0.041*
Motor blockade N (%)				p = 0.397
	0	55 (93.4%)	124 (83.2%)	
	+	6 (6.6%)	24 (16.1%)	
	++	0 (0.0%)	1 (0.7%)	

Table 3, a comparison between Groups A and B, showed no difference in the pain scores at rest or at movement, or in need for rescue analgesia. Significant differences were found regarding the presence of motor blockade.

**Table 3:** Demographic characteristics and comparison between Group A and Group B. M: motor; R: rest; N: number; *: statistically significant; +: slight decrease in muscle strength; ++: marked decrease in muscle strength.

		Group A	Group B	
				p-value
N		205	61	
SEX (F/M)		144 (70.2%)/61 (29.8%)	45 (73.8%)/16 (26.2%)	p = 0.594
Age (years)				p = 0.3577
	40-49	2 (1.0%)	1 (1.6%)	
	50-59	30 (14.6%)	5 (8.2%)	
	60-69	73 (35.6%)	24 (39.3%)	
	70-79	82 (40.0%)	29 (47.5%)	
	80-89	18 (8.8%)	2 (3.3%)	
	>90	0 (0.0%)	0 (0.0%)	
Maximum pain R N (%)				p = 0.291
	0	123 (60.0%)	43 (70.5%)	
	1	74 (36.1%)	17 (27.9%)	
	2	8 (1.0%)	1 (1.6%)	
	3	0 (3.9%)	0 (0.0%)	
	4	0 (0.0%)	0 (0.0%)	
Maximum pain M N (%)				p = 0.383
	0	24 (11.7%)	8 (13.1%)	
	1	107 (52.2%)	31 (50.8%)	
	2	65 (31.7%)	22 (36.1%)	
	3	9 (4.4%)	0 (0.0%)	
	4	0 (0.0%)	0 (0.0%)	
Rescue analgesia (N/%)		30/14,6%	4/6,6%	p = 0.097
Motor blockade				p < 0.001*
	0	149 (72.7%)	55 (93.4%)	
	+	45 (22.0%)	6 (6.6%)	
	++	11 (5.4%)	0 (0.0%)	

We registered no postoperative complications in terms of infection, hematoma, or nerve injury in any group.

## Discussion

TKA is a frequently performed major orthopedic surgery that might cause severe pain in the postoperative period. The use of peripheral nerve blocks as an element of multimodal analgesia regimens for pain management after TKA has become increasingly common due to their ability to reduce postoperative pain and opioid consumption. However, postoperative muscle strength preservation is a critical factor in the rehabilitation process and concerns have been raised about the potential negative impact of nerve blocks and tourniquet usage on muscle function, inducing weakness and impairing postoperative mobilization. In this study, we aimed to explore the potential advantages of implementing a specific strategy to promote enhanced recovery after surgery by optimizing pain management and preserving the motor function of patients undergoing TKA. This study compared the analgesic efficacy and the extent of motor blockade resulting from a single shot SNB or an iPACK block for pain management after unilateral, primary TKA. Additionally, the study analyzed the impact of using a T/D during the surgical procedure on the reported pain scores and motor function of the patients.

Previous studies have shown that the combination of the iPACK block with the ACB has a better analgesic effect than the latter alone and is conducive to an early functional recovery of patients [15,16]. Et et al. concluded that the association of the iPACK and ACB resulted in a significantly shorter length of stay and time to mobilization than the ACB in isolation or periarticular infiltration with ACB [16]. Similar results were published by Sankineani et al. who reported that lower pain scores, better ambulation distances, and knee range of motion were found in ACB with IPACK block in comparison with ACB alone [15]. Analyzing Groups A and C, the results of our study showed a statistically significant motor-sparing capacity of the iPACK block when compared to the SNB, maintaining good analgesic properties. Despite patients with SNB having lower pain scores on movement, most patients with an iPACK block continued to have no pain or mild pain during movement (Table 1). Therefore, our findings suggest that the iPACK block may be an alternative to the more traditional nerve blocks, like the SNB, which frequently have a negative impact on functional recovery, and are no longer recommended for pain management following primary TKA. Our findings align with Hussien et al. who compared the analgesic effectiveness of ultrasound-guided combined femoral and SNB versus ACB + iPACK block in patients undergoing TKA and concluded that there was less motor power impairment in the ACB-iPACK group within the first 24 hours postoperatively and no significant difference in visual analogue pain score between groups [17]. Another study conducted by Zheng et al. reported higher quadriceps femoris muscle strength, lower modified Bromage scale, and higher walking distance scores in the ACB + iPACK group than in the femoral nerve block combined with the SNB group [18].

A comparison between Groups B and C unveiled lower pain scores during movement (p = 0.021) and reduced necessity for rescue analgesia (p = 0.041) in Group B. These findings align with prior studies suggesting that avoiding the usage of a tourniquet may potentially result in reduced postoperative pain and analgesic consumption [11]. Similar results were found by Concina et al. related to the use of postoperative drains, with suction drains associated with higher pain scores [19]. Additionally, these authors reported lower hemoglobin values and higher transfusion rates in patients submitted to TKA with postoperative drainage [19].

Comparing Groups A and B (Table 3), no significant differences in pain scores during movement (p = 0.383) or in the need for rescue analgesia (p = 0.097) were observed. In fact, the superiority observed in the analgesic efficacy provided by the SNB may have been attenuated by the decrease in pain experienced by patients who underwent surgery without T/D. Although the non-use of a tourniquet may show some advantages, it is worth mentioning that tourniquet application is associated with lower perioperative blood loss and operation time [20,21].

This study is retrospective in nature and possesses several other limitations. The use of a qualitative pain scoring system relies on the patient’s subjective experiences of pain, which a range of factors, such as mood, anxiety, and cultural background can influence. These scores may also be influenced by some evaluation bias from healthcare providers that may result in inaccurate pain score recordings, making it less than ideal to compare two interventions. Additionally, the peripheral nerve blocks and catheter placements conducted by different anesthesiologists might have introduced variability in the approach. Furthermore, the performance of nerve blocks following spinal anesthesia could have influenced the assessment of block effectiveness. The use of different doses of dexamethasone (4 or 8 mg IV) may have had some influence on the nerve block duration and, subsequently, on the registered patient’s pain scores. Also, we discuss the utilization of rescue analgesia but do not detail the total morphine consumption, as this could have influenced the reported pain scores. Finally, we did not collect data on blood loss, drop in hemoglobin levels, reoperations, durability of prosthesis, or cement thickness which could be important to extract more information on the influence of T/D in TKA.

## Conclusions

This study’s findings indicate that the combination of the iPACK block with cACB for pain management following TKA can facilitate early mobilization and rehabilitation exercises after surgery without a significant negative impact on postoperative muscle strength. Moreover, this approach, cACB + iPACK without T/D, offers an effective analgesic outcome compared to the combination of cACB with popliteal SNB. This study also suggests that the use of T/D may be a source of postoperative pain, increasing the need for rescue analgesia and potentially compromising early rehabilitation after TKA.

The obtained results indicate that implementing an enhanced recovery program, including motor-sparing nerve blocks, and adopting surgical techniques without the use of T/D may benefit patients undergoing TKA, reducing pain and allowing a faster rehabilitation process. However, further research is necessary to validate these results and assess the long-term effects and surgical outcomes of such an approach.
